# Reprogramming alternative macrophage polarization by GATM-mediated endogenous creatine synthesis: A potential target for HDM-induced asthma treatment

**DOI:** 10.3389/fimmu.2022.937331

**Published:** 2022-09-13

**Authors:** Li Yu, Lingwei Wang, Guang Hu, Laibin Ren, Chen Qiu, Shun Li, Xiaohui Zhou, Shanze Chen, Rongchang Chen

**Affiliations:** ^1^ Department of Pulmonary and Critical Care Medicine, The First Affiliated Hospital (Shenzhen People’s Hospital) and School of Medicine, Southern University of Science and Technology, Shenzhen, China; ^2^ Department of Animal Model, Shanghai Public Health Clinical Center, Fudan University, Shanghai, China

**Keywords:** macrophage, polarization, creatine, glycine amidinotransferase (GATM), asthma

## Abstract

Cellular energy metabolism plays a crucial role in the regulation of macrophage polarization and in the execution of immune functions. A recent study showed that Slc6a8-mediated creatine uptake from exogenous supplementation modulates macrophage polarization, yet little is known about the role of the *de novo* creatine *de novo*biosynthesis pathway in macrophage polarization. Here, we observed that glycine amidinotransferase (GATM), the rate-limiting enzyme for creatine synthesis, was upregulated in alternative (M2) polarized macrophages, and was dependent on the transcriptional factor STAT6, whereas GATM expression was suppressed in the classical polarized (M1) macrophage. Next, we revealed that exogenous creatine supplementation enhanced IL-4-induced M2 polarization, confirming recent work. Furthermore, we revealed that genetic ablation of GATM did not affect expression of M1 marker genes (Nos2, IL1b, IL12b) or the production of nitric oxide in both peritoneal macrophages (PMs) and bone marrow-derived macrophages (BMDMs). By contrast, expression levels of M2 markers (Arg1, Mrc1, Ccl17 and Retnla) were lower following GATM deletion. Moreover, we found that deletion of GATM in resident alveolar macrophages (AMs) significantly blocked M2 polarization but with no obvious effect on the number of cells in knockout mice. Lastly, an upregulation of GATM was found in lung tissue and bronchoalveolar lavage fluid macrophages from HDM-induced asthmatic mice. Our study uncovers a previously uncharacterized role for the *de novo* creatine biosynthesis enzyme GATM in M2 macrophage polarization, which may be involved in the pathogenesis of related inflammatory diseases such as an T helper 2 (Th2)-associated allergic asthma.

## Introduction

Macrophages, which are professional phagocytes, are particularly plastic and dynamic during a variety of inflammatory responses (1). To adapt to complex environmental changes, macrophages can acquire distinct functionally polarized phenotypes regardless of the tissue of origin (2). Of note, upon stimulation by lipopolysaccharide (LPS) and/or interferon-γ (IFN-γ), macrophages polarize to the classically activated phenotypes (M1), which have anti-microbial properties *via* the production of effectors such as nitric oxide and defensins, and amplify pro-inflammatory responses *via* by releasing cytokines and chemokines such as tumor necrosis factor-a (TNF-α), IL-1b, IL-12, IL-6, CXCL1, CXCL2, CXCL9, CCL2, and CCL3 (3, 4). It is well documented that the essential transcription factors involved in regulating the expression of M1-related genes are nuclear factor kappa beta (NF-κB), signal transducer and activator of transcription 1/2 (STAT1/2), and interferon regulatory factor-1/3/5/7(IRF1/3/5/7) (5–9). In contrast, T helper 2 (Th2)-cell-associated cytokines such as interleukin-4/13 (IL-4/13) promote the alternative polarized macrophage phenotype (M2) (4), which is characterized by induction of the anti-inflammatory cytokine IL-10, transforming growth factor β (TGF-β), vascular endothelial growth factor (VEGF), arginase 1 (Arg1), resistin-like-α (Retnla) and macrophage mannose receptor1 (Mrc1). This allows M2 macrophages to execute functions that encourage tissue repair and resolve inflammation in addition to participating in Th2-associated chronic inflammatory disease progression (10, 11). M2 polarization is controlled by different transcription factors such as Krüppel-like factor 4 (KLF4), IRF4, STAT6, peroxisome proliferator-activated receptor γ (PPARγ), and C/EBPβ (12–14). Therefore, clarifying the regulatory molecular mechanism of macrophage polarization will be helpful for interpreting the pathogenesis of inflammatory diseases and finding potential drug intervention targets.

An accumulative body of evidence indicates that macrophage polarization is firmly coupled together with altered cellular energy metabolism (15, 16). Metabolic transitioning between glycolysis and oxidative phosphorylation (OXPHOS) is associated with M1 and M2 polarization processes, respectively (17). For example, during M1-polarization-related glycolysis under anaerobic conditions, glucose converts into pyruvate, and further converts to lactate, which is controlled by various glycolytic enzymes including hexokinase (HK), phosphoglucose isomerase (PGI), phosphofructokinase (PFK), aldolase, and pyruvate kinase. Notably, pharmacological inhibition of glycolysis with 2-deoxy-D-glucose (2-DG) blocks pro-inflammatory genes expression during M1 polarization (18). In contrast, M2 polarization is dependent on oxidative phosphorylation (OXPHOS), which is tightly coupled to the TCA cycle driven by acetyl coenzyme A (acetyl-CoA), pyruvate, fatty acids and α-ketoglutarate (αKG) reactions (19). Pu-Ste Liu et al. reported that the production of α-ketoglutarate (αKG) *via* glutaminolysis is important for alternative (M2) macrophage activation (20). Therefore, modulation of metabolic pathways are vital for macrophage polarization.

The arginine-creatine metabolic axis is thought to play a crucial role in macrophage polarization. Creatine is synthesized from glycine for the backbone, arginine for the amidine group, and S-adenosyl methionine (SAM) which supplies the methyl group. Its synthesis is catalyzed by amidinotransferase and methyltransferase. Creatine kinase catalyzes the transfer of sodium from ATP into the creatine molecule to form phosphocreatine reserves (21). Notably, L-arginine has various metabolic pathways available. For example, L-arginine is converted to NO by inducible nitric oxide synthase (iNOS) and is also utilized by arginase 1, two enzymes which are hallmarks of M1 and M2 polarized macrophages, respectively, and it is also converted to creatine by two enzymes, glycine amidinotransferase (GATM) and guanidinoacetate methyltransferase (GAMT) (22). Creatine, a widely used dietary supplement, was first described in 1835 by the French chemist Eugene Chevreau (23). Most endogenous creatine is produced naturally in the liver and kidneys. Creatine can be phosphorylated to creatine phosphate by creatine kinase, which serves as a phosphate donor in the conversion of ADP to ATP, and thus supplies energy necessary for cellular needs in an acute manner such as during muscle contraction (24). It has been reported that creatine impacts inflammatory responses. For example, Madan and Khanna observed in the late 1970s that creatine can ameliorate carrageenan-induced inflammation of the foot pads in rats (25). Deminice et al. demonstrated that TNF-α and CRP levels were lower in individuals supplemented with creatine following acute exercise (26). Creatine can also be obtained from the food and taken up by Slc6a8-expressing cells. Recently, Ji et al. reported that macrophages express relatively high levels of Slca68, and specific deletion of Slc6a8 in macrophages significantly reduce the amount of macrophage creatine and increases the expression of the M1 markers iNOS and CXCL9, whilst decreasing expression of the M2 marker Arg1 (22). This demonstrates that Slc6a8-mediated exogenous creatine uptake plays an essential role in macrophage polarization. In previous work from our group, we used microarray and RNA-sequencing and surprisingly observed that macrophages expressed both creatine *de novo* synthesis enzymes, GATM and GAMT, which was dramatically induced polarization in M2 but suppressed it in M1, indicating that the mechanism underlying macrophage polarization is likely *via* metabolic reprogramming.

For the most part, research on creatine biology has focused on the brain, muscle and the liver (27). The function of creatine in the immune system still largely unknown. In present study, we investigated the role of the rate limiting enzyme GATM in macrophage polarization. We found that GATM is required for polarization of M2 but not M1. The expression of GATM was positively associated with M2 macrophages in the BALF of HDM-induced asthma model mice. The current study implicatesGATM-mediated endogenous creatine synthesis as a key player in macrophage metabolism and opens a new avenue for possible treatment targets in macrophage-related inflammatory diseases such as asthma.

## Materials and methods

### Animals and establishment of a mouse model with asthma

Balb/c mice (6–8 weeks; GemPharmatech Co., Ltd, China) were sensitized by injections (s.c.) of HDM extract (50 μg in 100 μL sterile saline; Greer Laboratories, lot XPB82D3A25) on days 0, 7, and 14, followed by daily intranasal (i.n.) rechallenges (50 μg in 20 μL sterile saline) on days 21, 22, K23, 24, and 25. Nonsensitized mice received sterile saline only (s.c and i.n). Analysis of lung function parameters and organ collection was performed 24 h after the last challenge. All measures were taken to keep animal suffering to a minimum.

The CRISPR/Cas9 design target was designed on exon3 of the transcript GATM-201, causing frame shift mutations to achieve the purpose of inactivating protein function (**Figure S1A**). GATM-sgRNA1 targeting CCAAAGATCATCTGAAGAAGG was used for microinjection. We used PCR to identify GATM mouse genotypes (**Figure S1B**). The GATM knockout F0 generation was obtained by embryo transfer and surrogate conception, and the F1 generation was bred to determine the GATM genotype using T7E1 and sequencing (**Figure S1C**). The pure heterozygotes were further determined using Sanger sequencing results. Mice were housed in a specific pathogen-free animal facility and maintained in isolated ventilated cages on a 12-h light/dark cycle with ad libitum access to food and water.

### Lung histopathology

Tissue was taken from the left lung of each mouse and fixed in 4% paraformaldehyde overnight. The tissues were embedded in paraffin and then cut into 4-μm sections. Lung tissues were stained with H&E, periodic acid-Schiff (PAS), and Masson’s trichrome to observe histological changes including inflammatory-cell infiltration into the peribronchial connective tissues, mucus secretion and fiber formation.

### BALF analysis

After blood was taken, the lungs were lavaged *in situ* twice with 0.8 mL sterile saline (0.9% NaCl, prewarmed), and the recovered fluid was pooled. The total number of cells in BALF were counted, and a cytospin sample was prepared. The number of total blood cells, eosinophilic granulocytes, and lymphocytes in BALF were evaluated using a hemacytometer. The BALF was centrifuged at 3000 × rpm at 4°C for 15 min to collect the supernatant. Levels of IL-4 in cell-free BALF were determined using ELISA kits according to manufacturer instruction (ELM-IL-4-1, Raybio).

### Measurement of serum IgE

Mice were sacrificed with an overdose of pentobarbital (100 mg/kg, i.p.) one day after the last airway challenge. Blood samples were taken from the retroorbital plexus/sinus, allowed to rest at room temperature for 1 hour, then centrifuged (3000 × rpm, 20 min), and supernatants were collected for detection of total IgE with an ELISA kit according to manufacturer instructions (ELM-IgE-1, Raybio).

### Airway hyperresponsiveness measurement

AHR was measured using an animal pulmonary function instrument (Buxco Electronics, US). In brief, an ascending series of methacholine (6.26-50 mg/mL) was administrated into the trachea through the connected atomizer. The baseline airway resistance was evaluated using atomized PBS. The resistance index (RI) of the total lung and airway was determined as per the protocols of the instrument.

### Immunohistochemical staining

For GATM immunohistochemistry, deparaffinized lung sections were subjected to antigen retrieval, and then treated with H_2_O_2_ for 15 min to block endogenous peroxidase, and then incubated overnight at 4°C in recommended dilutions of anti-GATM antibodies. After washing with PBS, slices were incubated with a secondary antibody for 20 min at room temperature. Signals were visualized with DAB.

### Immunofluorescence staining

The lung tissue was fixed in 4% paraformaldehyde for 1 hour at 4°C, blocked in 20% goat serum for 2 hours at 4°C, and incubated overnight with the antibodies against GATM (GB113430, Servicebio), CD68 (GB113109, Servicebio), and Arginase 1 (GB11285, Servicebio) at 4°C, and then with fluorescein isothiocyanate-labeled secondary antibody (GB22303, Servicebio), at room temperature for 1 hour. We then used a fluorescent triple staining kit (G1236-100T, Servicebio). In addition, DAPI (G1012, Servicebio) was used to stain the nuclei and anti-fluorescence quenching sealer (G1401, Servicebio). The staining was observed under an inverted fluorescence microscope (Servicebio).

### Extraction and culture of alveolar macrophages, peritoneal macrophages and bone marrow-derived macrophages

Primary AMs were isolated by repeatedly flushing mouse lungs with 1 ml of PBS eight times at room temperature to obtain BAL. Cells were centrifuged at 1,500 rpm for 5 min, washed twice using DMEM complete medium, and then cells were incubated for 3 hours in 24-well plates at a density of 3 x 10^5^ cells/well to allow cells to adhere. Next, the cells were washed twice with PBS to remove non-adherent cells for subsequent processing. To generating peritoneal macrophages, intraperitoneal injections of 3% thioglycolate broth were administered, and then cells were extracted and harvested for assays. To generate BMDMs, bone marrow cells were cultured in macrophage complete medium containing 20 ng/ml M-CSF (CB34, Novoprotein). After 6 days in culture, non-adherent cells were eliminated and adherent cells were harvested for assays. Next, the AMs, PMs and BMDMs were cultured in 1 μg/ml LPS (Sigma-Aldrich, St. Louis, MO, USA) and 20 ng/ml IFN-γ (C746, Novoprotein) for M1 polarization, or 20 ng/mL interleukin-4 (CK15, Novoprotein) for M2 polarization treatment for up to 24 hours. M1 and M2 macrophages were harvested for quantitative PCR and western blotting analysis.

### RNA interference

For RNA interference experiments, primary cells were seeded in a 24-well plate in antibiotic-free growth medium. siRNA transfection was performed using Lipofectamine RNAi MAX reagent (13778150, Invitrogen) and Opti-MEM (Gibco, Germany) according to the manufacturer protocol. The sequences for siGATM (RIB-BIO; China) and siSTAT6 (RIB-BIO; China) were GGTCGAAGAGATGTGCAAT and GCTGATCATTGGCTTTATT, respectively; both used at a 50 nM concentration.

### Purification of CD4^+^ T cells from the mouse spleen

Primary BMDMs were incubated in 12-well plates (2 x 10^5^ cells/well) for 48 h to allow siRNA to interfere with GATM. To purify CD4^+^ T cells from the mouse spleen, spleens were dissected from healthy, female C57BL/6 mice (6–8 weeks old) under sterile conditions. A 100 μm cell strainer was placed in a sterile 10-cm dish and lymphocytes were isolated using a mouse lymphocyte separator (Dakewe, China) and CD4^+^ lymphocyte subsets were enriched on T-cell columns (Miltenyi, Germany) by negative selection according to manufacturer instructions and red blood cells lysed with ACK lysis buffer.

### Co‐culture of macrophages with CD4^+^ T cells

Mouse BMDMs and purified CD4^+^ T cells were seeded into 12‐well plates at a density of 2 × 10^5^ cells/well in lower room and 2×10^6^ cells/well and the upper room. Anti-mouse CD3 0.5 mg/l (eBioscience, America) and anti-mouse CD28 0.5 mg/l (eBioscience, America) antibodies were also added into the co‐culture system to stimulate the proliferation of CD4^+^T cells. After 72 hours of co‐culture, T cells were collected, and quantitative PCR was used to detect T-bet and IL-10.

### RNA isolation and quantitative PCR analysis

RNA isolation from AMs, PMs, BMDMs were performed *via* using the Total RNA extraction Kit (B511361-0100, BBI, China). Resulting total RNA was transcribed using *via* a HiScript^®^ III RT SuperMix cDNA Synthesis kit (R223-01, Vazyme, China) according to the manufacturer instructions. Quantitative PCR was performed using *via* ChamQ Universal SYBR quantitative PCR Master Mix (Q711-02, Vazyme, China) on a CFX Connect™ Real-Time System from BIO-RAD (BIO-RAD). β-actin was used as a reference gene. The sequences of primers used were as follows:

mouse β-actin-F TCCATCATGAAGTGTGACGT
mouse β-actin-R GAGCAATGATCTTGATCTTCAT
mouse Nos2 F CCTGTGAGACCTTTGATG
mouse Nos2 R CCTATATTGCTGTGGCTC
mouse TNF-α F CACCACGCTCTTCTGTCT
mouse TNF-α R GGCTACAGGCTTGTCACTC
mouse IL1b F CAACCAACAAGTGATATTCTCCATG
mouse IL1b R GATCCACACTCTCCAGCTGCA
mouse IL12b F GGAAGCACGGCAGCAGAATA
mouse IL12b R AACTTGAGGGAGAAGTAGGAATGG
mouse Arg1 F GGAACCCAGAGAGAGCATGA
mouse Arg1 R TTTTTCCAGCAGACCAGCTT
mouse Mrc1 F CATGAGGCTTCTCCTGCTTCT
mouse Mrc1 R TTGCCGTCTGAACTGAGATGG
mouse Ccl17 F TTGTGTTCGCCTGTAGTGCATA
mouse Ccl17 R CAGGAAGTTGGTGAGCTGGTATA
mouse Retnla F CGAGTAAGCACAGGCAGT
mouse Retnla R CCAGCTAACTATCCCTCCAC
mouse GATM-F TGAAGACAAGGCCACCCATC
mouse GATM-R GCATTTTCAGCTCTGCCCAC
mouse GAMT-F TTCCCTTGAAAGGCCTGTGG
mouse GAMT-R ACGTGAGGTTGCAGTAGGTG
mouse Slc6a8-F AGATCTCCGTTGCTCTGC
mouse Slc6a8-R TGGAAGACGTACATTCCACCATC
mouse Scd1-F GCTCTACACCTGCCTCTTCG
mouse Scd1-R GCCGTGCCTTGTAAGTTCTG
mouse T-bet-F TGTTCCCAGCCGTTTCTACC
mouse T-bet-R GCTCGGAACTCCGCTTCATA
mouse IL-10-F AGGCGCTGTCATCGATTTCT
mouse IL-10-R ATGGCCTTGTAGACACCTTGG
mouse IL-4-F ACGAGGTCACAGGAGAAGGGA
mouse IL-4-R AGCCCTACAGACGAGCTCACTC
mouse IL-5-F CTGGCCTCA AACTGGTAATGTAG
mouse IL-5-R ATGAGGGGGAGGGAGTATAACTC
mouse IL-13-F CCTCTGACCCTTAAGGAGCTTAT
mouse IL-13-R CGTTGCACAGGGGAGTCT

### Western blotting assay

Total protein was extracted using a protein extraction kit (CST, USA). The protein concentration was determined by a BCA Protein Assay kit (Beyotime, China). Cells were lysed for 10 min at 95 °C and 20 μg of protein was loaded onto 10% SDS-PAGE gel, then the separated proteins were transferred to a PVDF membrane (Merck Millipore, Bedford, MA, USA) and incubated overnight at 4 °C with primary rabbit monoclonal antibodies against iNOS (1:1000; 13120, CST, USA), rabbit monoclonal antibodies against Arginase-1 (1:1000; 93668, CST, USA), rabbit polyclonal antibodies against GATM (1:1000, PA5-109756, Invitrogen, USA), rabbit monoclonal antibodies against STAT6 (1:1000; 5397, CST, USA), and β-actin-HRP(1:3000; AB2001, ABWays), followed by anti-rabbit-IgG horseradish peroxidase-conjugated secondary antibodies (CST, USA). Signals were detected by enhanced chemiluminescence (Merck Millipore, Bedford, MA, USA) and exposed using a ChemiDoc™ MP Imager (Bio-Rad, USA). The integral optical density of each sample was measured using Image J.

### Quantification of nitric oxide

Nitric oxide was detected using the Griess Reagent System (Promega, USA).

### Flow cytometry

Mouse lung tissues were ground into single cell suspensions using a Miltenyi automatic tissue grinder (Miltenyi, Germany). The flow cytometry antibodies used were CD11c (117309, Biolegend, USA) and Siglec F (155509, Biolegend, USA). Cell surface staining was performed at 4°C for 30–40 min in the dark. The purity of sorted populations was >99%, unless otherwise indicated. Data were obtained on a FACSCanto II clinical flow cytometry system (BD Biosciences) and analyzed using FlowJo software (FlowJo LLC, Ashland, Oregon).

### Creatine assay

30 mg of lung tissue was lysed using CelLytic™ M (sigma, USA), centrifuged at 12000 rpm for 10 min, and the supernatant was collected for detecting 570 nm absorbance, according to the manufacturer instructions for Creatine colorimetric/fluorometric assay kit (BioVision, USA).

### RNA-seq analysis

Total RNA was extracted from AMs and PMs from WT and GATM^-/-^ mice using BBI reagent kits (BBI, China). High quality total RNA was used to construct an RNA-seq library. AMs and PMs were cultured in 12-well plates with 500,000 cells/well. PMs were stimulated with LPS and IFN-γ or IL-4 for 24 h. The integrity and total amount of RNA were assessed using the RNA Nano 6000 Assay Kit from the Bioanalyzer 2100 system (Agilent Technologies, CA, USA). RNA-seq library preparation (AMPure XP system) was performed at Novogene, where sequencing was performed on an Illumina NovaSeq 6000 machine.

### Statistics and software

Data were analyzed using GraphPad Software (Prism 8.00, San Diego, CA, USA). Each experiment was performed independently in at least 3 times. Quantitative data are presented as mean ± S.D., according to the number of comparison groups, and Student’s *t*-tests or one-way ANOVAs were performed as required. *P <*0.05 was considered statistically significant. *P <0.05; **P <0.01; ***P <0.001; ****P <0.0001.

## Results

### Creatine promotes IL-4 induced-M2 polarization *in vitro*

**Figure 1:**
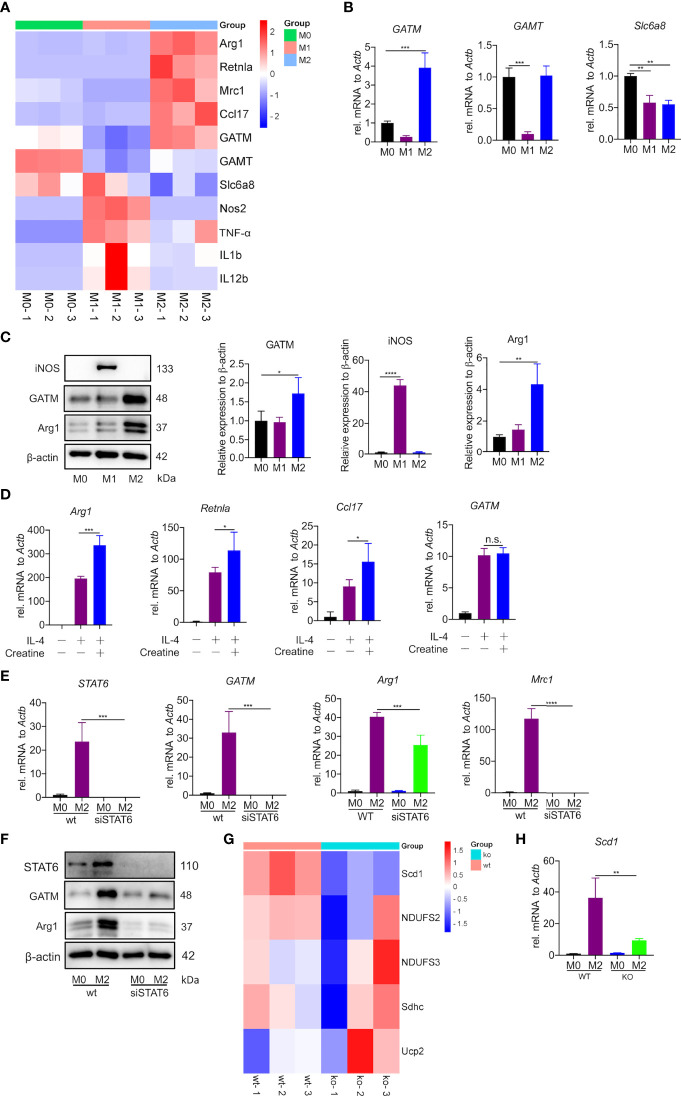
Creatine promotes IL-4 induced-M2 polarization *in vitro*. **(A)** Heatmap analysis of RNA-seq dataset of PMs showing subsets of genes in M1 and M2 macrophages relative to controls treated with LPS/IFN-γ or IL-4 for 24 hours. **(B)** Transcription levels of GATM, GAMT and Slc6a8 in BMDMs were detected by quantitative PCR (n=3). **(C)** Arg1 and GATM expression levels in BMDMs were detected using western blotting (n=3). **(D)** WT BMDMs were pretreated with creatine overnight and subsequently activated with IL-4 for 24 hours. Expression of Arg1, Retnla and Ccl17 mRNA were determined by quantitative PCR (n=4). **(E)** siRNA blocks STAT6 expression in IL-4 activated (24 hours) AMs. STAT6, GATM, Arg1 and Mrc1 expression was determined by quantitative PCR and **(F)**, western blotting. **(G)** Heatmap analysis of RNA-seq dataset of PMs showing subsets of genes in WT and GATM^-/-^ (n=3). **(H)** Transcription levels of Scd1 in AMs were detected by quantitative PCR (n=3). Data are expressed as mean ± SEM. Statistical significance was determined by one-way ANOVAs with Tukey’s multiple comparisons test; n.s., not significant *P<0.05, **P<0.01, ***P<0.001, ****P<0.0001.

The creatine transporter protein Slc6a8 is known to activate M2 polarization by transporting exogenous creatine (22). However, the role of endogenous creatine synthesis (GATM) on macrophage polarization remains unknown. Here, to establish the macrophage polarization model, AMs, PMs and BMDMs were treated for 24 h either with LPS and IFN-γ to induce M1polarization or with IL-4 to induce M2 polarization. It is well known that DNA microarray technology is an established technique for profiling gene expression at the whole genomic level. To investigate potential polarization-relevant genes, we previously performed an Illumina microarray analysis of M1- and M2-polarized AMs (28). As expected, a great number of genes were found to be altered by polarization conditions; among these genes, we identified that GATM together with the M2 marker genes (Arg1, Mrc1, Ccl17 and Retnla) were clearly upregulated in M2-polarized AMs. In this study, we used RNA-sequence technology, which is well established for profiling gene expression of PMs at the whole transcription level, we found a decrease in GATM expression in M1 polarized macrophages. This indicates that M1 activation is negatively regulated by GATM, and is elevated in M2 polarized macrophages (**Figure 1A** and **Table S1**). Of the three genes associated with the synthesis and transport of creatine (GATM, GAMT, Slc6a8), quantitative PCR and western blotting analysis showed that only the expression levels of GATM were increased following M2 polarization, indicating that GATM is involved in M2 polarization (**Figures 1B, C**). To determine the effect of creatine on M2 polarization, we added creatine to PMs and quantitative PCR analysis showed elevated expression levels of Arg1, Retnla and Ccl17, whereas there was no change in GATM expression (**Figure 1D**). To further explore the regulatory factors in GATM expression, we used RNAi technology to knockdown STAT6, which a key transcription factor of M2 polarization, and we found that GATM mRNA and protein expression was reduced in IL-4 polarized macrophages (**Figures 1E, F**). Furthermore, we analyzed lipid-metabolism-related genes through transcriptome data and found that GATM knockout significantly reduced Scd1 expression (**Figure 1G** and **Table S1**). GATM knockdown also reduced Scd1 mRNA levels in M2 macrophages (**Figure 1H**). Taken together, we conclude that GATM acts as an endogenous proponent of M2 polarization and that GATM expression is regulated by STAT6. In addition, GATM knockdown inhibited lipid metabolism in M2 macrophages to some extent.

### GATM promotes M2 polarization in BMDMs, but has only a small effect on M1 polarization

**Figure 2:**
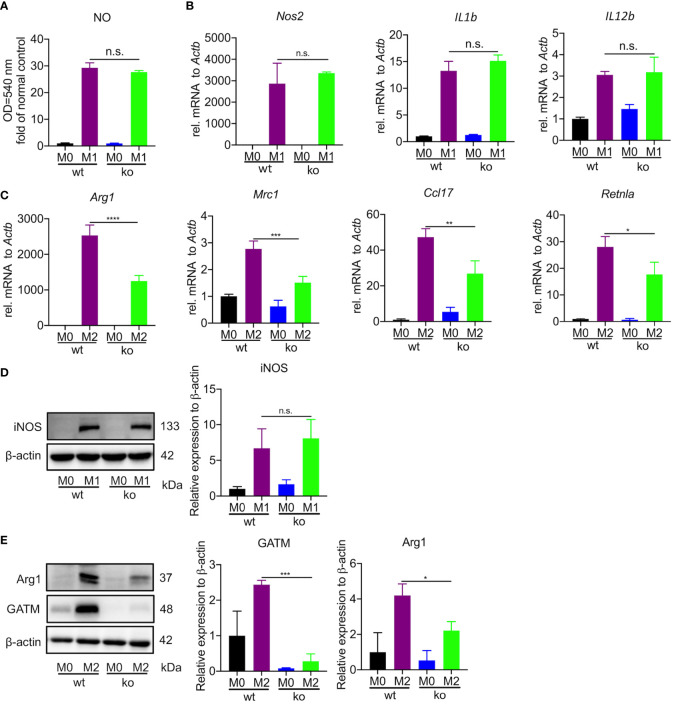
GATM promoted M2 polarization in BMDMs but had only a small effect on M1 polarization. **(A, B)**, GATM^-/-^ BMDMs were activated with LPS and IFN-γ for 24 hours. Level of NO and Nos2, IL1b and IL12b mRNA expression was determined by quantitative PCR (n=3). **(C)** GATM^-/-^ BMDMs were treated with IL-4 activation for 24 hours. Arg1, Mrc1, Ccl17 and Retnla mRNA expression was determined by quantitative PCR (n=3). **(D, E)**, GATM^-/-^ AMs were treated with LPS and IFN-γ or IL-4 activation for 24 hours. iNOS and Arg1 protein expression was determined by western blotting (n=3). Data are expressed as mean ± SEM. Statistical significance was determined using one-way ANOVAs with Tukey’s multiple comparisons test; n.s., not significant; *P<0.05, **P<0.01, ***P<0.001, ****P<0.0001.

To specifically address the role of GATM in macrophage polarization, we generated mice with GATM ablation and obtained GATM^-/-^ mice *via* CRISPR/Cas9-mediated gene editing. We found that GATM expressed in M2 polarization (**Figure S2**) and genetic ablation of GATM did not alter the level of nitric oxide (**Figure 2A**) and did not change transcript levels of Nos2, IL1b and IL12b (**Figure 2B**) or protein expression of iNOS (**Figure 2D**) in BMDMs. However, the expression of Arg1, Mrc1, Ccl17 and Retnla following IL-4-induced M2 polarization was reduced in GATM^-/-^ mice (**Figure 2C**) and protein expression of GATM and Arg1 (**Figure 2E**) was also reduced. Therefore, the results indicate that GATM had only a small effect on M1 macrophages, however, it did promote M2 polarization.

### AMs from GATM^-/-^ mice have altered expression of genes involved in cell-cell adhesion, immune response, cytokines and cytokine-receptor interactions and inhibit M2 polarization

**Figure 3:**
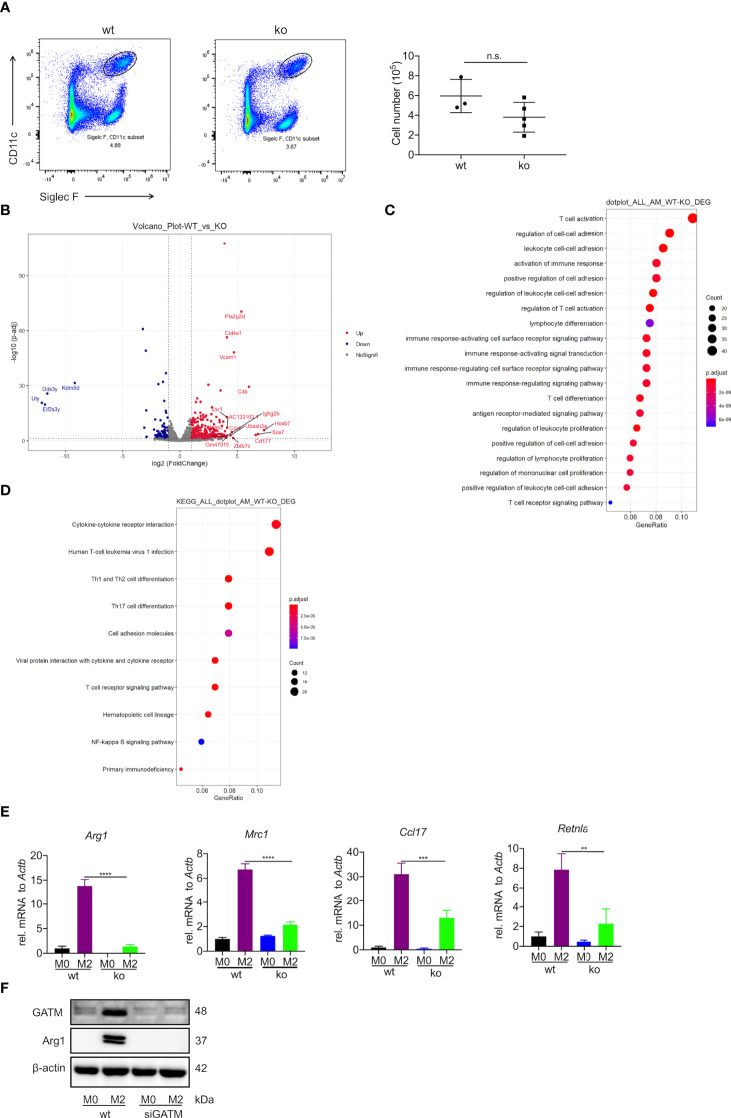
Deficiency of GATM inhibits M2 polarization in AMs. **(A)** AMs maturation was detected by flow cytometry (n=3–5). **(B)** Volcano plot depicting GATM knockdown upregulation of significantly upregulated genes. Red dots represent significant upregulation of expressed genes and blue dots represent significant downregulation of expressed genes (GATM^-/-^ vs. control) **(C)** Biological processes and **(D)** cell-signaling pathways related to AMs. Rich factor represents the number of differential genes/the total number of genes in the enrichment term; the greater the Rich factor, the higher the degree of GO/KEGG enrichment. Dot size represents the number of differential genes and the color of the dots represents the p-value in the enrichment analysis. siRNA blocks GATM expression in IL-4 activated (24 hours) AMs, **(E)** GATM and Arg1 protein expression was determined using western blotting (n=3). Data are expressed as mean ± SEM. Statistical significance was determined using one-way ANOVAs with Tukey’s multiple comparisons test; n.s., not significant **P<0.01, ***P<0.001, ****P<0.0001. **(F)** and inhibit M2 polarization.

Creatine is a metabolite involved in cellular energy homeostasis, which facilitates the differentiation of muscle cells and neurons (29, 30). To explore the contribution of GATM in the differentiation and maturation of AMs, we used flow cytometry to analyze the well-known AM markers CD11c and Siglec F. We observed a slight decrease in the number of AMs compared to wild type (**Figure 3A**). Furthermore, we used RNA-seq to compare the difference in transcriptomic analysis between wild type and GATM^-/-^ AMs. In the comparative transcriptomic analysis, a total of 434 DEGs, including 349 up- and 85 down-regulated genes, were identified from comparing data between the WT and GATM^-/-^ groups (**Figure 3B** and **Table S2**). We then used the DEGs for the bioinformatics analysis, which included Gene Ontology (GO) enrichment, Kyoto Encyclopedia of Genes and Genomes (KEGG) enrichment, and Ingenuity Pathway Analysis (IPA), to delineate the mechanism underlying the effect of GATM on macrophage polarization. In the comparison of WT and GATM^-/-^ groups, the GO enrichment analysis showed that the GATM deletion altered multiple biological processes related to T-cell activation, regulation of cell-cell adhesion and activation of immune response, (**Figure 3C**; **Table S3**). The KEGG pathway analysis highlighted cytokine/cytokine-receptor interaction, T-cell differentiation and viral-protein interaction with cytokines and cytokine receptors (**Figure 3D**; **Table S4**). We therefore investigated whether macrophage-intrinsic GATM dampens pro-inflammatory T-cell responses using *in vitro* co-culture of BMDMs with splenocytes. After 3 days of co-culture, GATM knockdown in BMDMs distinctly promoted the expression of T-bet and IL-10 compared to CD4^+^ T cells (**Figure S3**). IL-10 production by Treg cells is critical for the control of immune response and increases allergy-induced lung inflammation and hyperreactivity in Il10^flox/flox^×Foxp3^YFP-Cre^ mice (31). T-Bet expression acquisition by Tregs is necessary for the control of type 1 inflammation *in vivo (* 32). Furthermore, we found that GATM^-/-^ inhibited the expression of Arg1, Mrc1, Ccl17, Retnlain AMs (**Figure 3E**), and reduced the expression of Arg1 protein after GATM interference with AMs using RNAi technology (**Figure 3F**). Taken together, our results suggest that GATM promoted M2 polarization and altered clusters of functional genes involved in the immune response.

### Pulmonary expression of GATM and creatine is elevated in a HDM-induced asthma model

**Figure 4:**
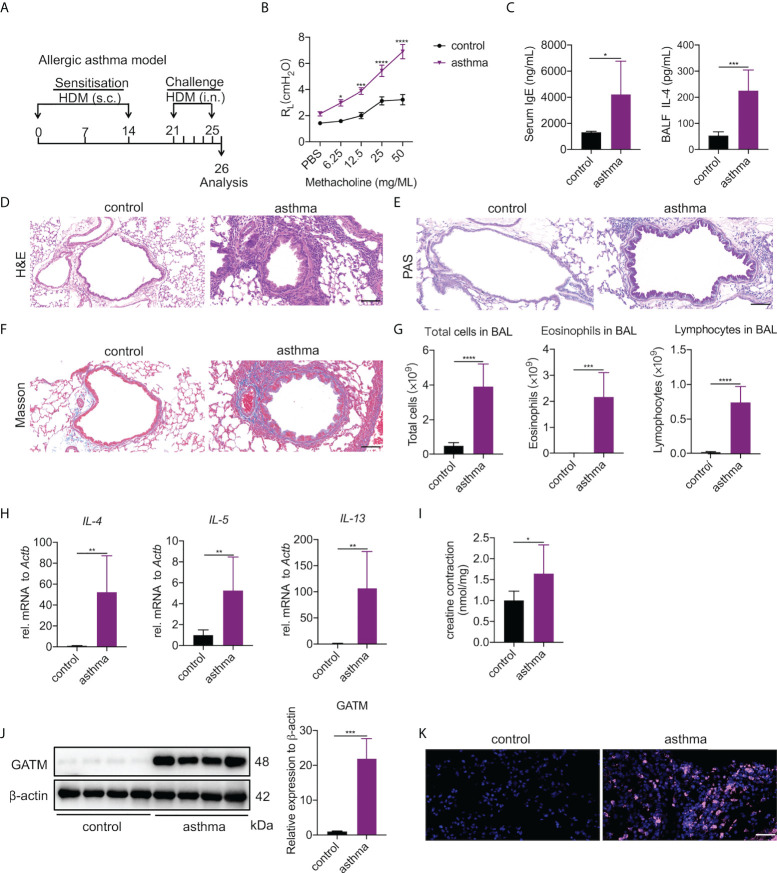
Pulmonary expression of GATM and creatine in HDM-induced asthma model. **(A)** Experimental timeline. **(B)** AHR as measured by lung resistance (R_L_; n=7). **(C)** Serum level of IgE and BALF levels of IL-4 (n=6–8). **(D)** Representative H&E-stained lung sections showing different groups. Original magnification x 200 (n=6). Scale bar=100 uM. **(E)** Representative PAS-stained lung sections showing goblet cell metaplasia in different groups. Original magnification x 200 (n=4). Scale bar=100 uM. **(F)** Representative Masson-stained lung sections showing submucosal collagen deposition in different groups. Original magnification x 200 (n=4). Scale bar=100 uM. **(G)** Total cells, eosinophils and lymphocytes in BALF (n=6). **(H)** The expression of IL-4, IL-5, IL-13 in asthma. **(I)** Creatine concentration in asthma. **(J)** western blotting detection of GATM protein expression in the lung (n=4). **(K)** Immunofluorescence detection of GATM protein expression in the lung (n=4). Scale bar=20 uM. Data expressed as mean ± SEM. Statistical significance was determined using t-tests; *P<0.05, **P<0.01, ***P<0.001, ****P<0.0001.

Asthma is a typical Th2-associated inflammatory disease in which macrophages exhibit M2 polarization (33). To link GATM expression with disease condition, we generated an acute HDM-induced murine model of asthma as a proxy for the acute inflammatory phenotype found in humans (34) (**Figure 4A**). As expected, airway exposure to HDM induced increased airway hyperresponsiveness to methacholine (**Figure 4B**), the majority of serum IgE and BAL IL-4 raised (**Figure 4C**). Examination of lung tissue revealed epithelium injury and smooth muscle thickening of the airway, and peribranchial inflammation with copious amounts of eosinophil and macrophage infiltration in the airway lumen were evident (**Figures 4D-F**). BAL cells had eosinophils and lymphocytes and other factors consistent with other HDM-induced asthma models (**Figure 4G**), indicating that the characteristics of chronic airway inflammation and airway remodeling are well demonstrated in this model. In addition, we detected that the transcriptional level of IL-4, IL-5 and IL-13 were significantly expressed in the lung tissue of asthmatic mice (**Figure 4H**). Studies have reported that creatine aggravates the inflammatory response in asthma (35, 36). Our results showed that the concentration of creatine in the lung tissue of asthmatic mice was higher than in the control group, indicating that creatine may be involved in the development of asthma (**Figure 4I**). Western blotting and immunohistochemistry of GATM showed that there was relatively high expression in lung tissue, and it appeared that HDM sensitization and challenge altered GATM expression in the lungs (**Figure 4J** and **Figure S4**). Consistent with this, immunofluorescence revealed that HDM exposure raised GATM expression across the whole lung (**Figure 4K**). Therefore, GATM is involved to some extent in the development of asthma.

### GATM is involved in the polarization of M2 type macrophages in asthma

**Figure 5:**
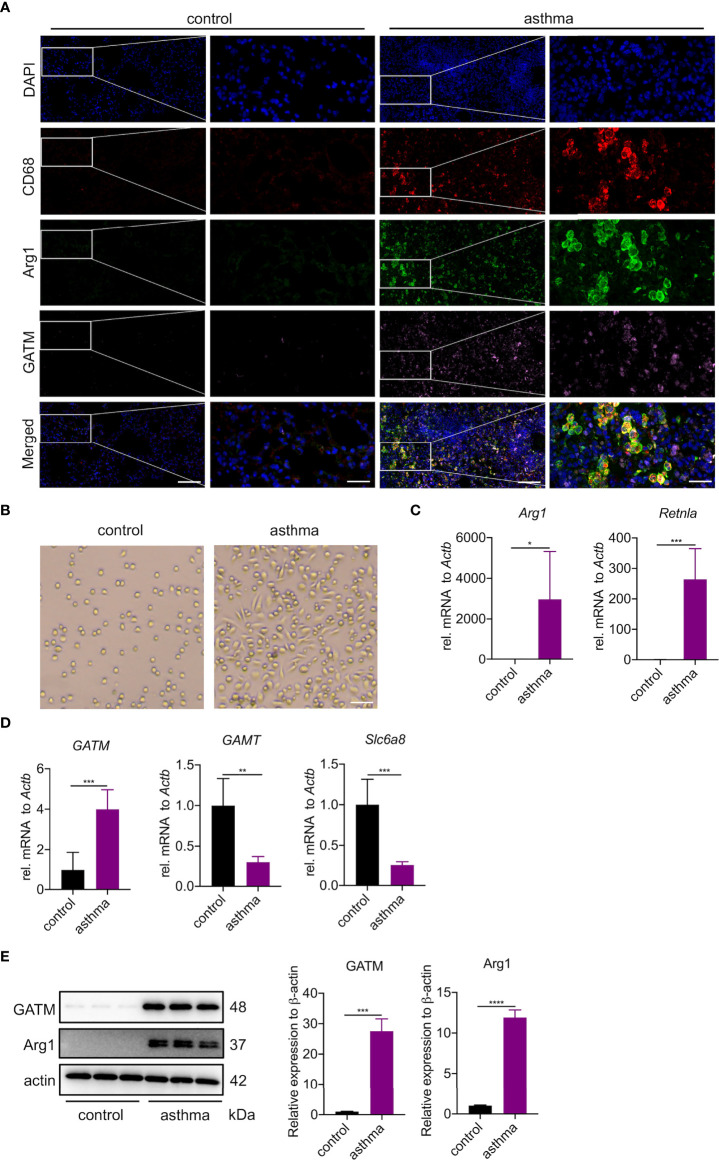
GATM is involved in the polarization of M2 type macrophages in asthma. **(A)** Immunofluorescence staining of CD68, Arg1 and GATM in lung tissue. Original magnifications x 200 *left* and x 630 *right* panel (n=4). Scale bar=20 uM. **(B)** Morphology of AMs under the microscope. Original magnification x 100 (n=5). Scale bar=200 uM. **(C)** Transcription levels of Arg1 and Retnla in AMs were detected by quantitative PCR (n=5). **(D)** Transcription levels of GATM, GAMT and Slc6a8 in AMs were detected by quantitative PCR (n=5). **(E)** Arg1 and GATM expression levels in AMs were detected by western blotting (n=4). Data are expressed as mean ± SEM. Statistical significance was determined using t-tests; *P<0.05; **P<0.01, ***P<0.001, ****P<0.0001.

To investigate whether the upregulated GATM in asthmatic lung tissue was related to macrophage polarization, we analyzed GATM colocalization with M1 (CD68, iNOS) and M2 (CD68, Arg1) markers in lung tissue using confocal microscopy, and found that GATM was upregulated in M2 macrophages but not M1 polarized macrophages (**Figure 5A** and **S4A**). Furthermore, we isolated AMs using BAL and cultured them for 3 hours to adhere to the well walls, then observed them under the microscope and found that that the majority of macrophages in the asthma group exhibited a shuttle-like shape, while the control group alveolar macrophages still retained a round shape (**Figure 5B**). We detected that mRNA expression of Arg1, Retnla and GATM was upregulated in AMs, but not in GAMT, Slc6a8, Nos2, IL1b, or IL12b (**Figures 5C, D** and **Figure S4B**). Moreover, western blotting verified the upregulation of GATM and Arg1 protein levels in these cells (**Figure 5E**). Overall, our present study suggests that GATM may contribute to the progression of asthma *via* modulation of M2 macrophage polarization.

## Discussion

In this study, we uncovered a novel GATM function in alternative macrophage activation, which is dependent on the STAT6 pathway. RNAi- and CRISPR/Cas9-mediated deletion of GATM resulted in lower expression of M2 marker genes such as Arg1, Mrc1, Ccl17 and Retnla in polarized M2 AMs, PMs and BMDMs, suggesting a vital role of GATM in the regulation of innate immune function. Previous work reported that Slc6a8 regulated creatine uptake and synthesis in PMs, indicating that Slc6a8 mediated macrophage metabolism in the resting state (22). In contrast, we found that Slc6a8 expression was reduced in polarized macrophages. However, GATM expression was increased in polarized M2 macrophages, but not in polarized M1 macrophages, indicating that GATM regulates M2 polarization. In addition, GATM was differently expressed in M1 and M2 polarized macrophages, indicating that it is likely to be regulated by disparate mechanisms. Furthermore, we found that STAT6 regulated GATM expression but we did not find direct evidence in the TFBS database for STAT6 regulation of GATM expression, probably for STAT6 indirectly regulating GATM expression through regulating intermediate molecules. Previous studies have shown that the addition of exogenous creatine promotes the expression of M2 markers (Arg1, Retnla) in PMs (22), and we also confirmed this result in BMDMs, suggesting that creatine promotes M2 polarization in macrophages of different origins using similar regulatory mechanisms. Moreover, we found that GATM^-/-^ mice had inhibited expression of Scd1, suggesting that GATM promoted M2 macrophage polarization by affecting lipid metabolism. Typically, studies on macrophages have used PMs and BMDMs as models. We also utilized BMDMs as a model and showed that GATM does not affect M1 polarization and that endogenous creatine has little effect on M1 polarization. Endogenous creatine promotes cancer metabolism through activation of Smad2/3 (37). Nevertheless, a large number of studies have shown that exogenous creatine inhibits M1 polarization *via* JAK-STAT signaling (22, 38), but not *via* NF-κB signaling. It is known that endogenous creatine and exogenous creatine have distinct roles and regulatory mechanisms on M1 polarization in diverse diseases. Previous studies have shown that exogenous creatine promotes M2 polarization *via* the STAT6 pathway (22), and here we demonstrated that both exogenous and endogenous creatine promote M2 polarization *via* the STAT6 pathway, which complements the immunological effects of endogenous creatine found in previous research. In addition, we also list other transcription factors that may regulate GATM expression in **Table S5**. For example, one study found that monounsaturated fatty acids generated by Stearoyl-CoA desatSurase-1 (SCD1), an enzyme responsible for the desaturation of saturated fatty acids, reduced the surface abundance of the cholesterol efflux transporter ABCA1, which in turn promoted lipid accumulation and induced an inflammatory phagocyte phenotype (39). We found that GATM knockdown robustly decreased the mRNA expression of Scd1 but not of Scd2 isoforms in AMs, indicating GATM knockout blocked creatine synthesis and inhibited lipid metabolism, thereby inhibiting M2 polarization. In addition, complementing the immune mechanism of action of endogenous creatin.

Macrophages are the only cells that are present in every organ in the body. They mainly have roles in anti-inflammatory responses and tissue repair (40), which have received much attention. We previously found that GATM was significantly expressed in M2 polarization, but the effect of GATM deletion on macrophage polarization remained uncertain. Therefore, we generated GATM^-/-^ transgenic mice using CRISPR/Cas9 technology and, despite decreasing the number of cells, did not affect the maturation of alveolar macrophages, likely because of the inconsistent number of mice, or because of individual differences. It may be that increasing the samples size would give different results in this instance. Macrophages and T cells activate and suppress each other and together regulate the environmental homeostasis of the organism (41). RNA-seq further analyzed GATM^-/-^ in AMs and found enrichment of T-cell activation and immune-related pathways, demonstrating that GATM^-/-^ may activate T-cell-mediated immune responses. In addition, GATM may be necessary for Tregs control of Th1 inflammation. The effect of GATM^-/-^ on AMs was consistent across PMs and BMDMs (reduced Arg1, Mrc1, Ccl17, Retnla), suggesting that GATM possibly affects M2 polarization through the same mechanism in both AMs and PMs. It has been suggested that Mrc1 (42, 43), Retnla (44–46) and Ccl17 (47–49) mediate allergic sensitization and asthma or COPD. Accordingly, GATM may be a good target for the relief of allergic inflammation.

GATM is the key rate-limiting step that catalyzes endogenous creatine biosynthesis: the transfer of amino groups from arginine to glycine to form ornithine and guanidinoacetic acid directly promotes creatine biosynthesis, which is supplemented by cytoplasmic ATP *via* phosphocreatine shuttling while consuming mitochondrial ATP (37). In hereditary diseases where creatine synthesis is impaired, creatine has a disease‐modifying capacity, including mental fatigue (50), sleep deprivation (51, 52), environmental hypoxia such as in mountain climbing and advanced age (53–55), but except people with kidney disease (56). Recent studies have found that exogenous creatine aggravates the inflammatory response in asthma (35), but the mechanism had not been elucidated. We found that GATM was significantly expressed in the mouse asthma model, but not GAMT and slc6a8. Therefore, we mainly explored the expression of GATM in macrophages. The expression of IL-4 was still significantly elevated in the HDM-induced asthma model. *In vitro*, we used IL-4 to stimulate M2 polarization. Based on this, we speculate that GATM expression increases in asthma may also be due to Th2-type immune-factor stimulation. However, due to homozygous infertility restrictions, our research did not adopt an acute HDM-induced GATM^-/-^ murine model of asthma to study the effect of creatine on the inflammatory response of asthma, which is limitation of this study.

## Data availability statement

The datasets presented in this study can be found in online repositories. The names of the repository/repositories and accession number(s) can be found in the article/**Supplementary Material**.

## Ethics statement

The animal study was reviewed and approved by The Animal Subjects Committee of Shenzhen People’s Hospital.

## Author contributions

LY, SC, and RC conceptualized and designed the study. SL, XZ, SC, and RC carried out the experiments. LW, CQ, RC, and XZ analyzed the data. LY, SC, and RC drafted the manuscript. LY, SC, and RC edited the manuscript for language. SL, XZ, SC, and RC revised the manuscript. All authors contributed to the article and approved the submitted version.

## Funding

This study was supported by grants from the National Natural Science Foundation of China (81803183, 82170042), the Shenzhen Science and Technology Innovation Program (JCYJ20210324114400002), the Natural Science Foundation of Guangdong Province (2022A1515011966), the Key Project of Science and Technology Foundation of Sichuan Province (2019YFS0230) and the Project of Shanghai Municipal Health Commission (No.20204Y0335).

## Conflict of interest

The reviewer ZJ declared a shared parent affiliation with the authors, WZ, SL, to the handling editor at the time of the review.

The remaining authors declare that the research was conducted in the absence of any commercial or financial relationships that could be construed as a potential conflict of interest.

## Publisher's note

All claims expressed in this article are solely those of the authors and do not necessarily represent those of their affiliated organizations, or those of the publisher, the editors and the reviewers. Any product that may be evaluated in this article, or claim that may be made by its manufacturer, is not guaranteed or endorsed by the publisher.

## Supplementary material

Supplementary Figure 1 GATM mouse construction protocol. (A) Analysis of conserved regions of mouse GATM gene structure and protein function. (B) GATM mouse genotyping PCR product. (C) Results of T7E1 analysis of GATM mouse genotyping.

Supplementary Figure 2 GATM expression in M2 polarized macrophages.

Supplementary Figure 3 The expression of T-bet and IL-10 was detected by quantitative PCR (n=3). Statistical significance was determined using one-way ANOVAs with Tukey’s multiple comparisons test; ***P*<0.01, ****P*<0.001, *****P*<0.0001.

Supplementary Figure 4 GATM expression in the HDM-induced asthma model using immunohistochemistry (n=4). Scale bar=50 uM.

Supplementary Figure 5 GATM is barely affected in M1 polarized macrophages in asthma. (A) Immunofluorescence staining of CD68, iNOS and GATM in lung tissue. Original magnifications x 200 *left* and 630 *right* panel (n=4). Scale bar=20 uM (B) The expression of Nos2, TNF-α, IL1b, IL12b was detected by quantitative PCR (n=5). Data are expressed as mean ± SEM. Statistical significance was determined using t-tests; **P*<0.05, ****P*<0.001, *****P*<0.0001.
