# Supplementary figures and images for: Reprogramming alternative macrophage polarization by GATM-mediated endogenous creatine synthesis: A potential target for HDM-induced asthma treatment
Source: Front Immunol. 2022 Sep 13;13:937331. doi: 10.3389/fimmu.2022.937331 (PMC9513582; doi:10.3389/fimmu.2022.937331)

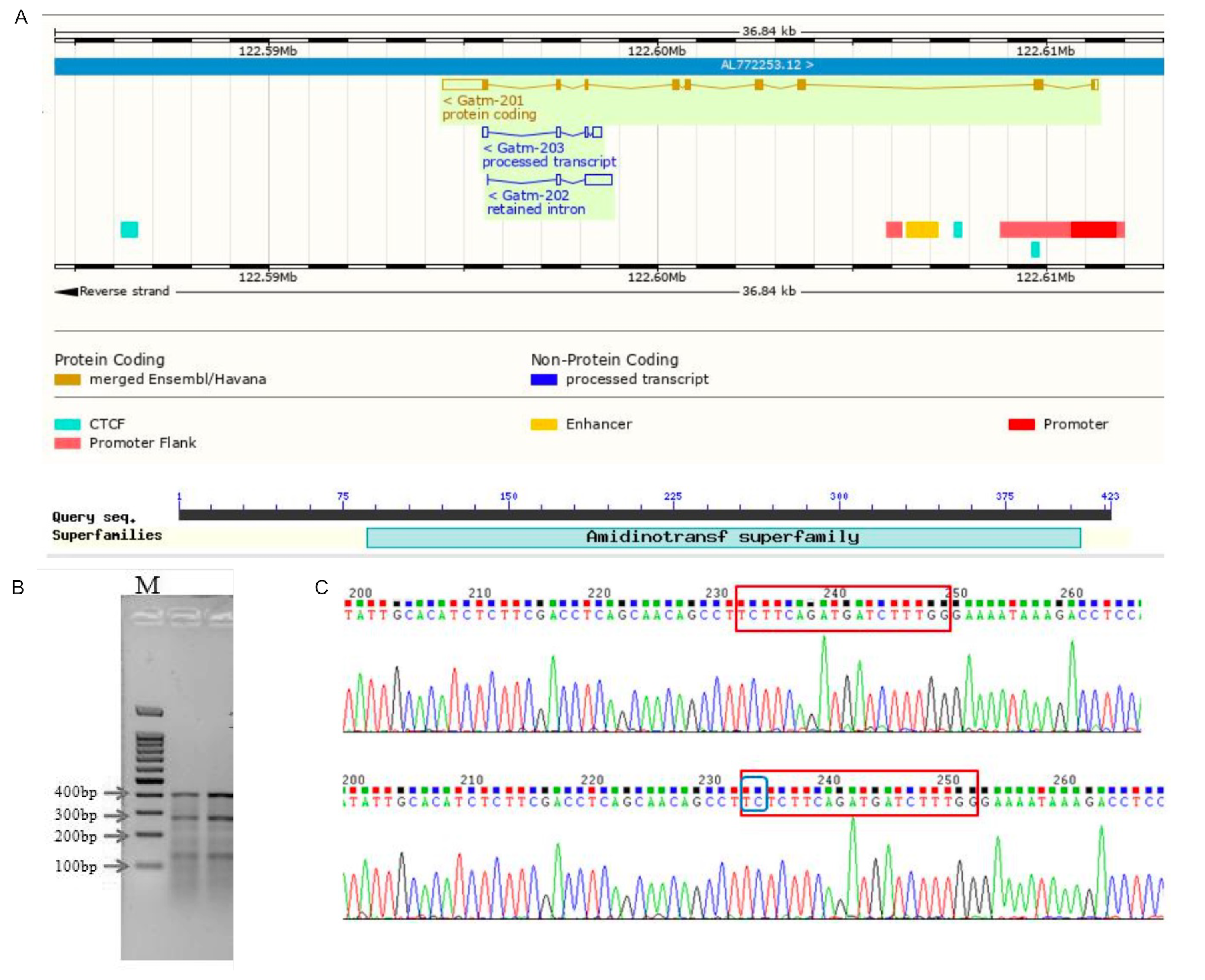

Supplement: Supplementary Figure 1 — GATM mouse construction protocol. (A) Analysis of conserved regions of mouse GATM gene structure and protein function. (B) GATM mouse genotyping PCR product. (C) Results of T7E1 analysis of GATM mouse genotyping. [file Image_1.tif]

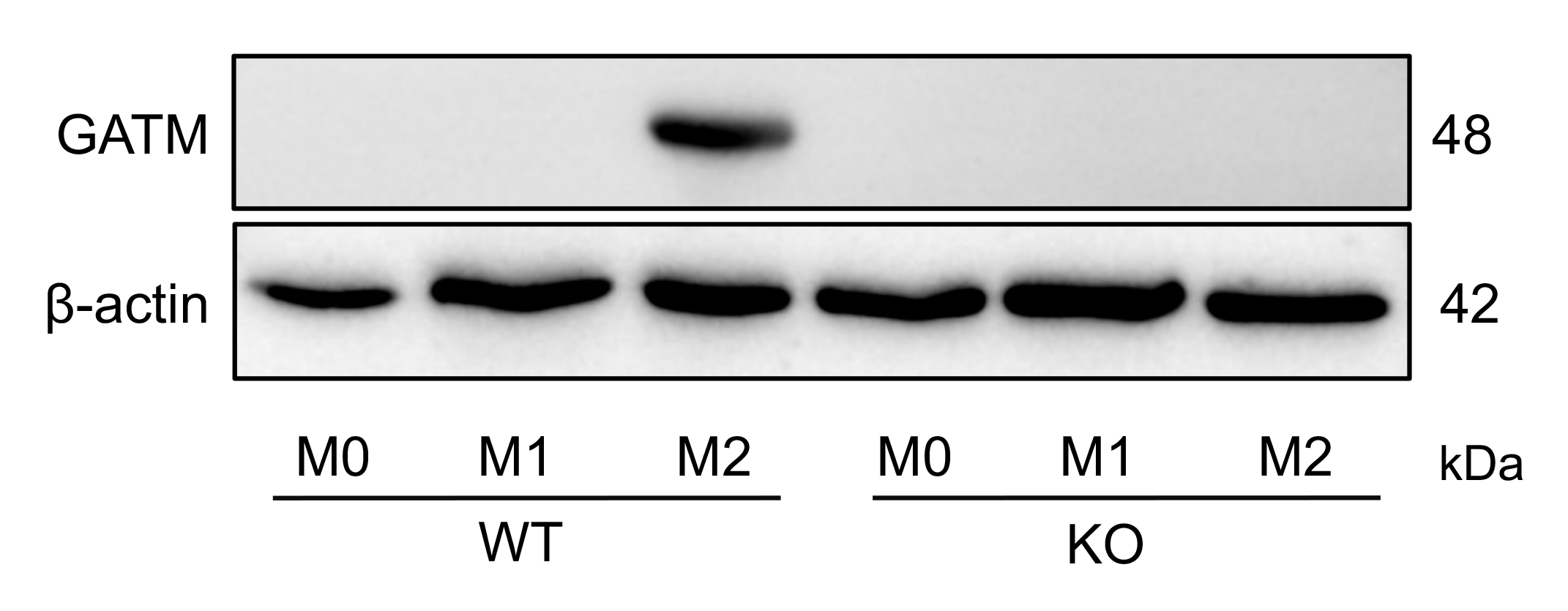

Supplement: Supplementary Figure 2 — GATM expression in M2 polarized macrophages. [file Image_2.tif]

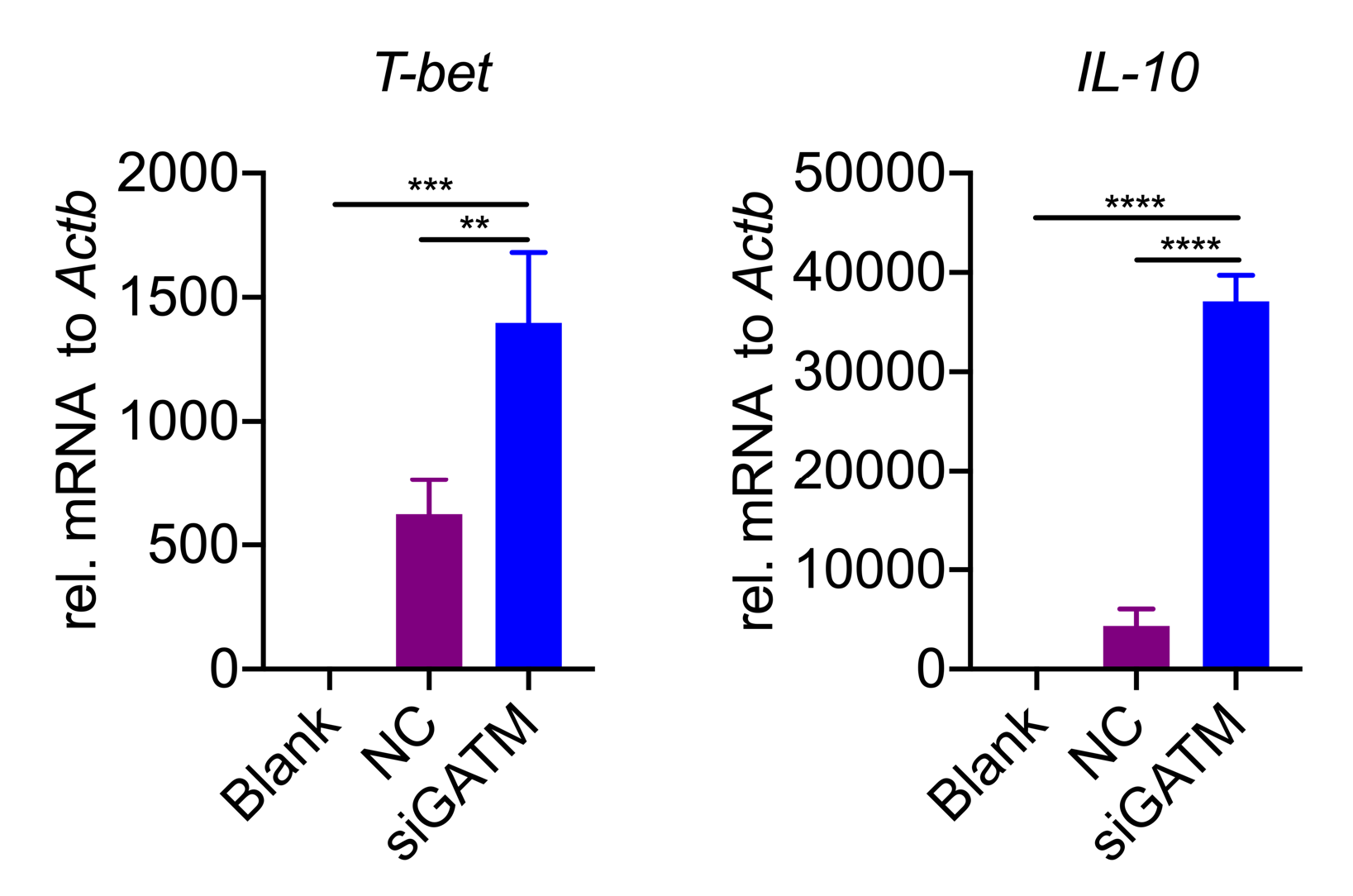

Supplement: Supplementary Figure 3 — The expression of T-bet and IL-10 was detected by quantitative PCR (n=3). Statistical significance was determined using one-way ANOVAs with Tukey’s multiple comparisons test; **P<0.01, ***P<0.001, ****P<0.0001. [file Image_3.tif]

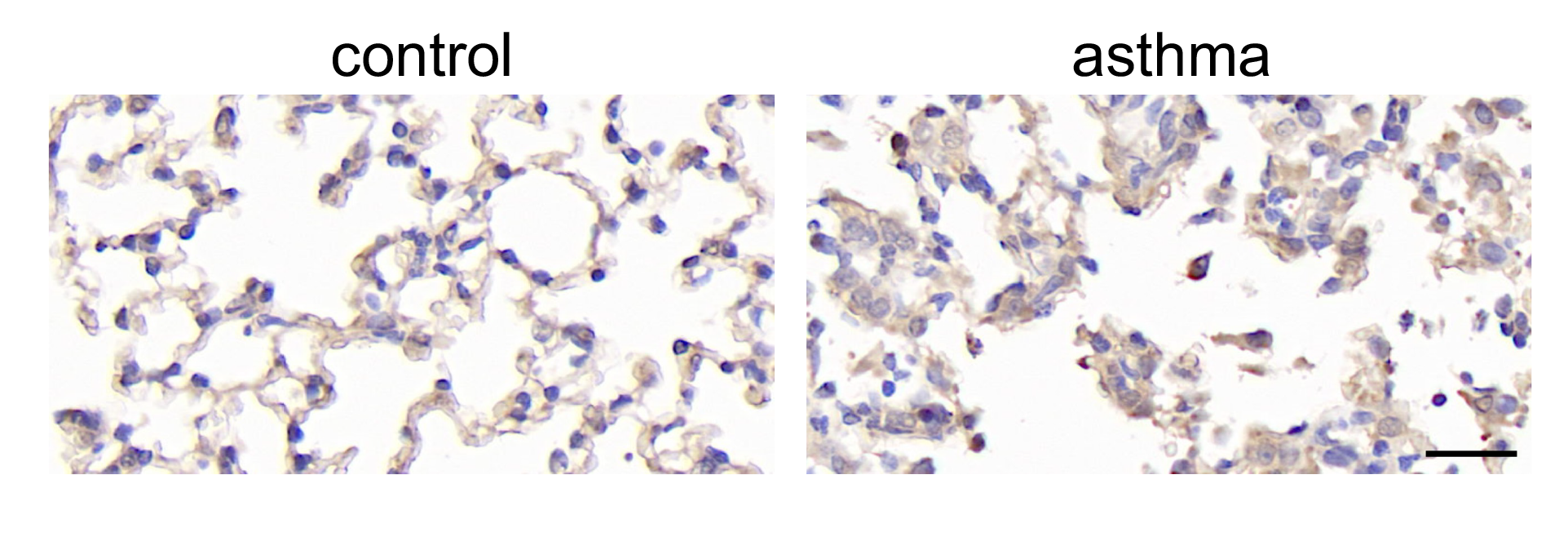

Supplement: Supplementary Figure 4 — GATM expression in the HDM-induced asthma model using immunohistochemistry (n=4). Scale bar=50 uM. [file Image_4.tif]

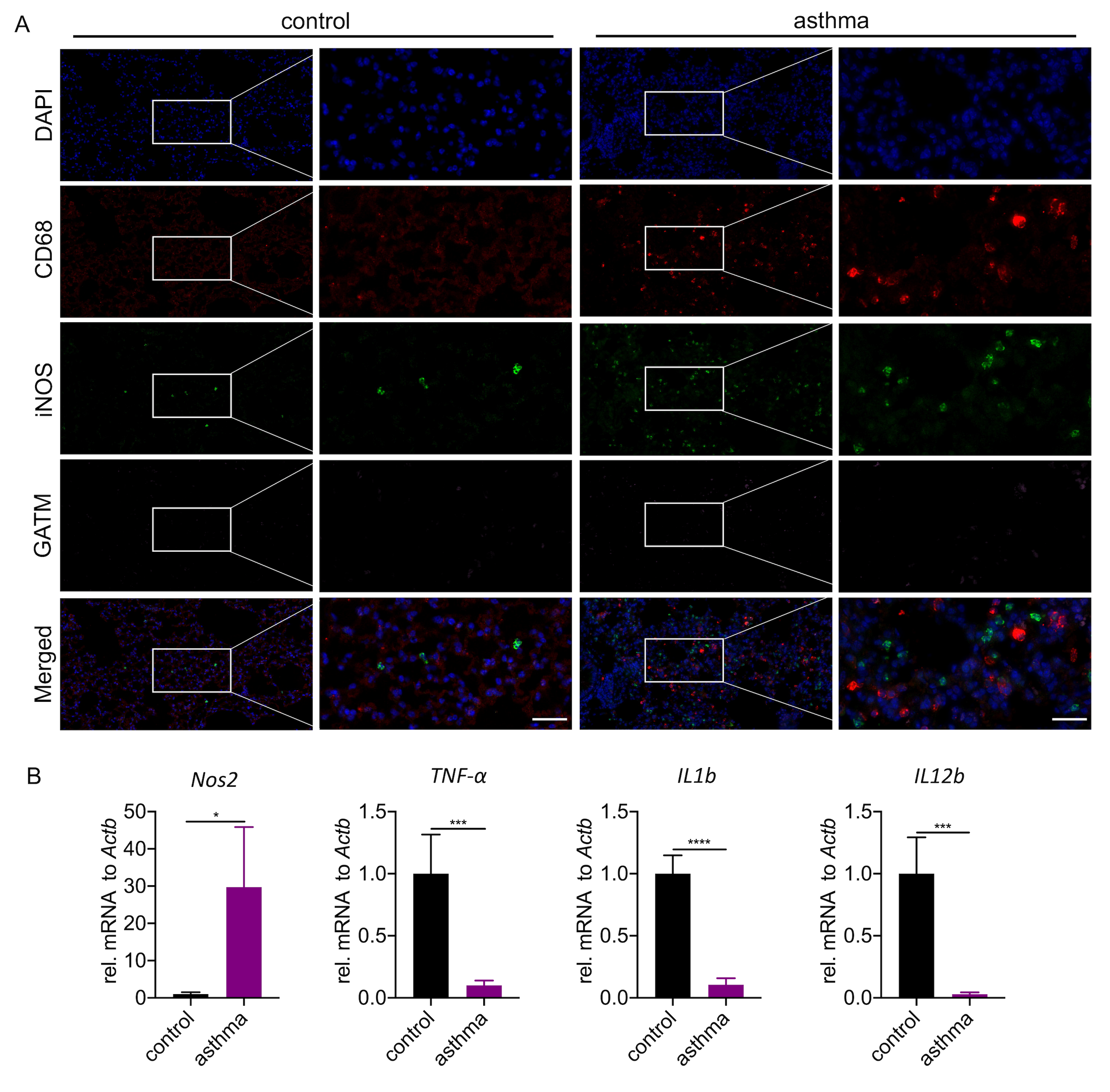

Supplement: Supplementary Figure 5 — GATM is barely affected in M1 polarized macrophages in asthma. (A) Immunofluorescence staining of CD68, iNOS and GATM in lung tissue. Original magnifications x 200 left and 630 right panel (n=4). Scale bar=20 uM (B) The expression of Nos2, TNF-α, IL1b, IL12b was detected by quantitative PCR (n=5). Data are expressed as mean ± SEM. Statistical significance was determined using t-tests; *P<0.05, ***P<0.001, ****P<0.0001. [file Image_5.tif]
